# Multimodal sensory evaluation of neuropathic spinal cord injury pain: an experimental study

**DOI:** 10.1038/s41393-020-00607-z

**Published:** 2021-01-14

**Authors:** Emmanuelle Opsommer, Natalya Korogod, Lenka Stockinger, Gunther Landmann

**Affiliations:** 1 School of Health Sciences (HESAV), University of Applied Sciences and Arts Western Switzerland (HES-SO), Lausanne, Switzerland; 2 Centre for Pain Medicine, Swiss Paraplegic Centre, Nottwil, Switzerland

**Keywords:** Neurology, Medical research, Chronic pain

## Abstract

**Study design:**

An experimental study.

**Objectives:**

To investigate the changes in somatosensory functions using the combined application of quantitative sensory testing (QST), contact heat-evoked potentials (CHEPs) and laser-evoked potentials (LEPs) studies in individuals with spinal cord injury (SCI) in relation to neuropathic pain (NeP).

**Setting:**

Centre for Pain Medicine, Swiss Paraplegic Centre, Nottwil, Switzerland.

**Methods:**

Individuals with SCI were compared: 12 with NeP (SCI NeP) and 12 without NeP (SCI no NeP). Tools used were QST, CHEPs, LEPs and self-reported questionnaires. Tests were applied to the control (hand) and test (dermatome of altered sensation) sites, and compared to the able-bodied group.

**Results:**

QST, LEPs and CHEPs assessments showed abnormalities both on the test and control sites, which did not differ between the groups with SCI. QST showed higher prevalence of allodynia in SCI NeP. CHEPs and LEPs demonstrated diminished amplitudes in both groups with SCI in comparison to able-bodied individuals. Only reaction time (RT) analysis revealed the difference of SCI NeP from the other two groups, expressed in partially preserved responses to the laser C-fibre stimulations.

**Conclusions:**

Combination of assessments in our study allowed to examine spinothalamic and dorsal column functions in individuals with SCI. Changes in QST, CHEPs and LEPs were detected below the level of injury independent of NeP and at the control site indicating modifications in sensory processing rostral to the spinal lesion. Analysis of RT during laser stimulation could be an essential component when evaluating the somatosensory functions related to NeP in persons with SCI.

## Introduction

Spinal cord injury (SCI) can be complicated by several disorders including pain, which have repercussions beyond the consequences of SCI. Persons with SCI often consider pain as their main problem [1]. They describe neuropathic pain (NeP) as very disturbing and find it even more intensive than musculoskeletal pain, which influence their daily living activities and quality of life [2, 3]. Multiple mechanisms are responsible for NeP development and chronification after SCI, which involve peripheral and/or central sensitisation related to the affected function of nociceptive terminals and fibres, gliosis and/or changes at the supraspinal level [4]. Particularly, central sensitisation, the condition that is maintained by sensitised C-fibres, and/ or disinhibition of the C-nociceptive system plays an important role [5].

Among methods existing to identify dysfunctions in the somatosensory system, the quantitative sensory testing (QST) represents one of the non-invasive procedures, which uses mechanical and thermal stimulations to assess function of Aδ, C and Aβ afferent fibres [6]. It was shown that QST, in combination with other tests, could be potentially used for both patient phenotyping [7] and NeP prediction in individuals with SCI [8].

Another objective alternative to QST, which is used for assessment of mainly Aδ-nociceptive pathways, is contact heat-evoked potentials (CHEPs). It was shown that in persons with traumatic SCI, CHEPs were missing in 94% of patients with below-level NeP and in 71% without NeP [9] and neither the presence nor the absence of CHEPs nor the latencies or amplitudes of the CHEPs allowed for differentiation between individuals with SCI and NeP, those without NeP and able-bodied people [9, 10].

Technical approaches used in CHEPs involve stimulation of relatively large skin areas, which does not allow testing Aδ- and C-fibres functions separately. Another technique, infrared laser-evoked potentials (LEPs) study, permits a more selective evaluation of Aδ- and C-fibres. Different conduction velocities of these afferents allow laser stimuli eliciting typical double sensation: an initial Aδ-fibre-related pricking pain followed by a C-fibre-related burning pain [11]. The brain responses elicited by such stimuli show components at latencies corresponding to Aδ- (late LEPs) [11] and C-fibres (ultra-late LEPs) [12]. Analysis of these components (shorter for Aδ- and longer for C-fibres) as well as recording the reaction time (RT) for each trial, where participant is requested to press a button as soon as the stimulus is perceived, allows distinguishing responses related to the Aδ- or C-fibres [12]. A few studies used late LEPs to investigate NeP in individuals with SCI. For example, Landmann et al. [13]. showed the usefulness of late LEP in combination with QST to detect lesions of the somatosensory system in that population. Vogel et al. [14] used these techniques to investigate the role of hypersensitivity in NeP development after SCI. Ultra-late LEPs are technically more difficult to record than late LEPs, only a few studies assessed their usefulness in patients with postherpetic neuralgia and depression [15, 16] but none in central NeP after SCI.

The aim of this experimental study was to investigate somatosensory dysfunctions using the combined application of QST, CHEPs and LEPs tests in individuals with SCI and NeP (SCI NeP) and to compare it to those without NeP (SCI no NeP) and to able-bodied participants.

## Methods

### Participants

Three groups each of 12 participants (11 men, 1 woman) aged between 18 and 65 years participated in this study: SCI NeP, SCI no NeP and able-bodied participants. Within the recruitment procedure (Supplementary Fig. 1), all patients diagnosed with SCI undergoing the yearly routine follow up between June 2015 and June 2016, which is performed for each patient at the Swiss Paraplegic Centre (Nottwil, Switzerland), were screened for the eligibility criteria. Inclusion criterion for both groups was traumatic SCI (>1 year) below T1 according to International Standards for Neurological Classification of Spinal Cord Injury [17] with a confirmed lesion by MRI or CT.

Additional inclusion criterion for the NeP group was at- or below-level NeP in the trunk or in the lower extremity. Exclusion criteria for all groups were any known neurological disorders that can interfere with the study, severe psychiatric disorders, metabolic problems, pregnancy, palliative care, other pain or not German speaking. Participants from all three groups were matched by age and sex; in addition both groups with SCI were also matched by lesion level and where possible with the American Spinal Injury Association (ASIA) Impairment Scale (AIS) grade [17]. Additional exclusion criteria for both groups with SCI were non-traumatic SCI, lesion level above T1 and no sensibility in the testing (painful) area. Participants with a complete SCI lesion (AIS grade A) with NeP were included only if the pain was present within the Zone of Partial Preservation (ZPP) [17] with impaired sensation. In participants with AIS grade B, C and D, all pain localisations below the neurological level of injury (NLI) were eligible for inclusion.

### Clinical examinations and self-reported questionnaires

For the SCI NeP, we followed clinical examination recommendations [4, 18, 19]. A neurologist performed careful analysis of the pain history and complete neurological examination including AIS grade and pain drawings done by the participant showing all pain sites [4]. Pain-related data such as pain character and the maximum intensity (on a 0–10 Numerical Rating Scale (NRS)) of each pain type were assessed. Each pain was classified [18, 19] and the certainty of NeP presence was graded [4].

The standardised German pain questionnaire, described in a previous study [13], was used. The psychological status was assessed with German version of the Depression Anxiety and Stress Scale (DASS) [20]. Health-related quality of life was measured using the EuroQol EQ-5D-3L questionnaire (German version) with the reference values for the Swiss population [21].

For pain history, we used tools described in a previous study [13]. In SCI NeP, chronic pain severity was assessed using the Graded Chronic Pain Scale (GCPS). The Mainz Pain Staging System (MPSS) defined the grade of chronicity of pain. In addition, neuropathic symptoms were measured using the German version of the Neuropathic Pain Symptom Inventory (NPSI) [22].

### Experimental procedure

The detailed experimental procedure is described in the Supplementary file. The examination sites for LEPs, CHEPs and QST were divided into a control site (clinically unaffected site) and a test site (corresponding to pain site of the NeP group). The control site in all three groups was dorsum of the hand (C6–C8). In able-bodied individuals, the test site was defined in the ventral thigh (according to the ASIA sensory spot L2). The test site for each participant with pain was defined during the neurological examination. In participants with a complete lesion (AIS A), the test site was chosen as the site of NeP within the ZPP. In participants with incomplete spinal lesions (AIS B, C and D), the test site was chosen according to the clinical pain presentation. In case of bilateral NeP, we chose site, where NeP was more intensive. The neurophysiological examinations were applied within the corresponding ASIA sensory spots. In SCI no NeP, the test site was chosen according to the matched SCI NeP participant.

### Quantitative sensory testing

QST was performed only for groups with SCI in accordance with the German Research Network on Neuropathic Pain (DFNS) protocol [6]. This protocol considers assessing all aspects of somatosensory function with 13 parameters for sensory loss and gain: thermal and mechanical parameters. The protocol and the tools used for testing each parameter have been described elsewhere [6, 13]. QST values were transformed to *z*-scores [6] and compared with normative values [23]. A score that was out of the 95% CI considered as a sensory abnormality with gain or loss function. Frequencies of abnormal QST parameters (in %) were calculated for both groups and sites and compared with expected values for able-bodied individuals (±2.5%) using one sample *t*-test.

### Laser-evoked potentials

Experiments were performed using a Thulium-YAG laser (StarMedTec GmbH, Starnberg, Germany), as described previously [13]. For the ultra-late LEPs measurements, we put the laser beam through a spatial filter (aluminium plate with thickness <1 mm) with a calibrated hole to reduce the diameter and obtain a tiny stimulation area (<0.25 mm²). The filter was placed close to the skin to reduce diffraction.

For LEPs measurements, laser sensory and pain thresholds were obtained using the method of levels as described elsewhere [13]. LEPs were recorded given a randomised application of two different laser intensities alternating randomly between 480 and 540 mJ for hand and trunk and 540–600 mJ for legs and feet. The inter-stimulus interval was randomised by the machine lasting between 7 and 15 s.

Ultra-late LEPs were recorded given a randomised application of laser intensities ranging between 840 and 900 mJ.

All evoked potentials were recorded from one Ag-AgCl scalp electrode at the vertex (Cz) based on the International 10–20 system with linked earlobes (A1–A2) as reference (band pass 1–30 Hz, sampling rate 500 Hz, impedance <5 kΩ) and a ground electrode attached to the right hand. The Electro-Oculo-Gram of the right eye was recorded from two linked surface electrodes.

Peak latencies of N2 and P2 components were identified and the peak-to-peak (N2 to P2) amplitudes were measured.

### Reaction time analysis

To evaluate the C-fibre-mediated responses by using ultra-late LEPs stimulation of the tiny skin area, participant had to perform a RT task consisting of pressing a button on a hand-controller as soon as he/she perceived any type of sensations at the stimulation site [12]. The RT distribution analysis was done, where following trials were distinguished: trials without response defined as ‘unperceived’ (RT = 0), trials with fast responses (corresponding to Aδ-fibres activations, between 200 and 600 ms post stimulus), trials with slow responses (corresponding to C-fibres activations, between 700 and 2500 ms post stimulus) and ‘error’ trials (faster than 200 ms and slower than 2500 ms).

### Contact heat-evoked potentials

For CHEPs, evoked potentials were recorded in response to skin stimulation using heat-foil thermode stimulator (Pathway, Medoc Ltd, Ramat Yishai, Israel) with a heating ramp of 70 °C/s. Cooling began immediately after the target heat temperature was achieved. The baseline temperature was 42 °C [24], destination temperature 52 °C, and stimulus interval ranged between 8 and 12 s. CHEPs were recorded and analysed similarly to LEPs.

### Statistical analysis

For data analysis, we used Igor Pro (WaveMetrics Inc., Version 6.37 for Windows, Portland, USA) and SPSS (IBM SPSS Statistics for Windows, Version 23.0. IBM, Armonk, NY, USA). A descriptive analysis was performed on demographic, clinical and SCI characteristics. Data were reported as means and standard deviations (SD), unless otherwise stated. Reaction time distributions were compared between test sites and groups using Kolmogorov–Smirnoff two sample test. Differences between the groups were calculated using *t*-test or one-way ANOVA for normally distributed values, but otherwise Mann–Whitney U test or Kruskal–Wallis test on ranks were used. Post-hoc analysis was done to identify the groups, where there was a statistical significance: Holm–Sidak method for one-way ANOVA and Dunn–Bonferroni for Kruskal–Wallis test. *P* value < 0.05 was considered statistically significant.

## Results

### Demographic and clinical characteristics of participants

During the period of the study, we screened 998 patients with SCI (Supplementary Fig. 1). After inspection of all exclusion criteria, we included 12 participants for both groups. Table 1 shows the summary of the clinical characteristics. Then, we recruited 12 able-bodied participants.

**Table 1:** Clinical characteristics of participants: participants with spinal cord injury with and without neuropathic pain and able-bodied people.

Characteristics	SCI NeP	SCI no NeP	Able-bodied	*P* value
Number (man, woman)	12 (11, 1)	12 (11, 1)	12 (11, 1)	
Age, years (mean ± SD)	45 ± 11	46 ± 14	46 ± 9	0.951^a^
Time since injury, years (mean ± SD)	10 ± 8	20 ± 13	–	0.039^b^
Neurological level			–	–
Thoracic	9	9		
Lumbar	3	3		
ASIA Impairment scale (AIS)			–	–
A	4	5		
B	0	0		
C	3	3		
D	5	4		
Lesion type			–	–
Spinal cord	7	5		
Spinal cord and Cauda equina	4	5		
Cauda equina	1	2		
Spasticity			–	–
Yes	9	7		
No	3	5		
EQ-5D VAS (mean ± SD)	66 ± 17	73 ± 17	89 ± 11	0.002^c^ (able-bodied-SCI NeP; able-bodied-SCI no NeP)
EQ-5D 3 L index (mean ± SD)	0.65 ± 0.17	0.75 ± 0.14	0.96 ± 0.08	<0.001^c^ (able-bodied-SCI NeP; able-bodied-SCI no NeP)
DASS				
Depression	5.6 ± 4.6	2.8 ± 2.6	1.3 ± 2.1	0.009^c^ (able-bodied-SCI NeP)
Anxiety	3.6 ± 3.5	1.4 ± 1.4	1.1 ± 2.0	0.052^a^
Stress	6.7 ± 4.2	4.2 ± 3.4	2.1 ± 4.3	0.03^a^ (able-bodied-SCI NeP; Able-bodied-SCI no NeP)
Medication		–		
Antiepileptic	5	–		
Opioids	1	1		
Antidepressants	1	–	–	–
Analgesics	2	2		
Baclofen	1	2		

*SCI* spinal cord injury, *NeP* neuropathic pain, *ASIA* American Spinal Injury Association, *EQ-5D* EuroQol EQ-5D-3L questionnaire, *VAS* Visual Analog Scale, *DASS* Depression Anxiety and Stress Scale.

^a^One-way ANOVA.

^b^Paired *t*-test.

^c^Kruskal–Wallis one-way ANOVA on ranks.

### Self-reported questionnaires

The outcomes of the self-administrated questionnaires for all three groups are reported in the Table 1 (DASS, EuroQoL). The global health level (EQ-5D VAS) and mean healthy utility EQ-5D index were within the normative range [21], but higher in able-bodied group in comparison to both groups with SCI. The results of DASS questionnaire showed that SCI NeP group had on average higher depression and stress levels than other groups, but within the normal values [20] (Table 1).

### Pain history for SCI NeP group

The pain-related variables for SCI NeP group are reported in the Table 2. All SCI NeP participants developed one or several out of four pain types [18]. Musculoskeletal pain in SCI NeP had about equal prevalence (5/12) as in SCI no NeP (6/12).

**Table 2:** Pain-related data for participants with spinal cord injury and neuropathic pain.

Characteristics	SCI NeP
Pain duration, years (mean ± SD)	8.9 ± 6.2
Pain intensity, NRS (according GCPS; 0–100, mean ± SD)	51 ± 19
Pain types	
(1) Nociceptive pain	
Musculoskeletal pain	6 (5)^a^
Visceral pain	0 (0)^a^
Other nociceptive pain	0 (0)^a^
(2) Neuropathic pain	
At level	8 (0)^a^
Below level	8 (0)^a^
Other neuropathic pain	0 (0)^a^
(3) Other pain	0 (0)^a^
(4) Unknown pain	0 (0)^a^
Number of pain types	
One pain type	3 (5)^a^
Two pain types	8 (0)^a^
Three pain types	1 (0)^a^
Pain site (NeP only)	
Trunk	2
Thigh	7
Foot	3
Measurements site in relation to pain	
At level	7
Below level	5
Pain descriptions (multiple announcement possible)	
(1) Hot-burning	4
(2) Tingling	1
(3) Pricking	3
(4) Pins and needles	4
(5) Sharp	1
(6) Shooting	1
(7) Squeezing	5
(8) Painful cold	0
(9) Electric shock-like	5
(10) Others	1
MPSS	
Stage I	3
Stage II	4
Stage III	5
GCPS	
Grade 1	6
Grade 2	4
Grade 3	0
Grade 4	2
Quality of neuropathic pain (NPSI)	
Total score (0–120), mean ± SD	40 ± 24
Sub scores NRS (0–10), mean ± SD	
Burning pain *n* = 9	5.2 ± 3.7
Pressure/squeezing pain *n* = 9	3.6 ± 3.5
Paroxysmal pain *n* = 10	5.2 ± 3.7
Allodynia *n* = 9	2.7 ± 2.3
Tingling/pins and needles *n* = 12	4.7 ± 3.5

*SCI* Spinal cord injury, *NeP* Neuropathic pain, *MPSS* Mainz Pain Staging System, *GCPS* Graded Chronic Pain Scale, *NRS* Numerical Rating Scale, *NPSI* Neuropathic Pain Symptom Inventory.

^a^n-number in the SCI no NeP group.

The area of pain was located at thigh region (*n* = 7), trunk (*n* = 2) and foot (*n* = 3). The pain was moderate (NRS 4–6). Half of the participants had low grade of chronic pain severity (Grade 1—GCPS) and about half the highest stage of pain chronification (Stage III—MPSS). According to NPSI questionnaire, 9/12 participants had burning, pressure/squeezing or allodynia symptoms, 10/12 had paroxysmal pain and all 12 had some tingling/pins and needles sensations. There was also a correlation between the total NPSI score and the level of stress (*r* = 0.537) and anxiety (*r* = 0.406).

### Quantitative sensory testing

Individual QST values, means and SD of *z*-scores for each parameter for both groups with SCI and sites are presented in Table 3 and Supplementary Fig. 2. Analysis of variance showed that QST parameters did not differ between the groups (except pressure pain threshold (PPT), but within the normative values), but did differ between the control (hand) and test (pain) sites and there was no interaction between group and site factors. More specifically, both groups with SCI showed significant loss (*p* ≤ 0.001) of function in cold detection threshold, warm detection threshold (WDT) and thermal sensory limen (TSL) on the test site in comparison to the control site and loss of function for vibration detection threshold (VDT) for both examination sites, with stronger expression on the test site. In addition, participants from SCI no NeP group had loss of function in mechanical detection threshold (MDT) at the test site.

**Table 3:** QST profile (*z*-scores) comparison between groups with SCI for the control and test sites.

	QST parameter Group.	CDT	WDT	TSL	PHS	CPT	HPT	PPT	MPT	MPS	DMA	WUR	MDT	VDT
Control site *z*-score	SCI NeP (mean ± SD, *n* = 12)	−0.365 ± 0.826	−0.351 ± 1.283	−0.389 ± 0.859	0(CI:0, 0)	1.542 ± 0.960	1.048 ± 0.785	0.200 ± 0.745	1.039 ± 0.577	0.018 (Cl: −0.528, 1.089)	0 (CI: 0, 0)	−0.105 (Cl: − 0.838, 0.265) (*n* = 5)	−1.697 ± 1.250	−2.026 ± 1.446
	SCI no NeP (Mean ± SD, *n* = 12)	−0.143 ± 1.138	−0.160 ± 1.501	−0.418 ± 1.282	0(CI:0, 0)	0.787 ± 1.116	0.056 ± 1.369	−0.806 ± 0.699	1.546 ± 1.110	0.547 (Cl: −0.064, 1.321)	0 (CI: 0, 0)	−1.135 (ch-i.ses, −0.163) (*n* = 5)	−1.606 ± 1.150	−2.053 ± 1.770
Test site *z*-score	SCI NeP (mean ± SD, *n* = 12)	−3.423 ± 1.902	−2.138 ± 2.093	−3.227 ± 1.703	0 (CI: 0,1)	−0.464 ± 0.885	−0.958 ± 0.876	−0.082 (CI: −0.863, 1.034) (*n* = 6)	−0.508 ± 1.318	−1.563 ± 2.445	0 (CI: 0, 0)	−0.891 (CI: −1.217, 0.879) (*n* = 5)	−1.872 ± 0.844	−3.486 ± 2.141
	SCI no NeP (mean ± SD, *n* = 12)	−3.050 ± 2.050	−3.122 ± 2.283	−3.199 ± 1.808	0(CI:0, 0)	−0.797 ± 0.828	−1.413 ± 0.473	−1.064 (CI: −1.827, 0.176) (*n* = 6)	−1.071 ± 1.953	−1.905 ± 2.414	0 (CI: 0, 0)	−1.762 (Cl: − 2.459, 0.446) (*n* = 5)	−2.803 ± 1.254	−2.722 ± 2.767
Two way ANOVA test *P* value	Group (1) SCI NeP vs. SCI no NeP	n.s.	n.s.	n.s.	n.s.	n.s.	n.s.	0.001	n.s.	n.s.	n.s.	n.s.	n.s.	n.s.
	Site (2) control vs. test	<0.001	0.001	<0.001	n.s.	<0.001	<0.001	n.s.	0.001	0.002	n.s.	n.s.	0.003	0.008
	(l) × (2)	n.s.	n.s.	n.s.	n.s.	n.s.	n.s.	n.s.	n.s.	n.s.	n.s.	n.s.	n.s.	n.s.

*SCI* spinal cord injury, *NeP* neuropathic pain, *CI* confidence interval, *QST* quantitative sensory testing, *CDT* cold detection threshold, *WDT* warm detection threshold, *TSL* thermal sensory limen, *PHS* paradoxical heat sensation, *CPT* cold pain threshold, *HPT* heat pain threshold, *PPT* pressure pain threshold, *MPT* mechanical pain threshold, *MPS* mechanical pain sensitivity, *DMA* dynamic mechanical allodynia, *WUR* wind-up ratio, *MDT* mechanical detection threshold, *VDT* vibration detection threshold, *n.s.* not significant, *n* number of participants concerned by the values.

**Fig. 1:**
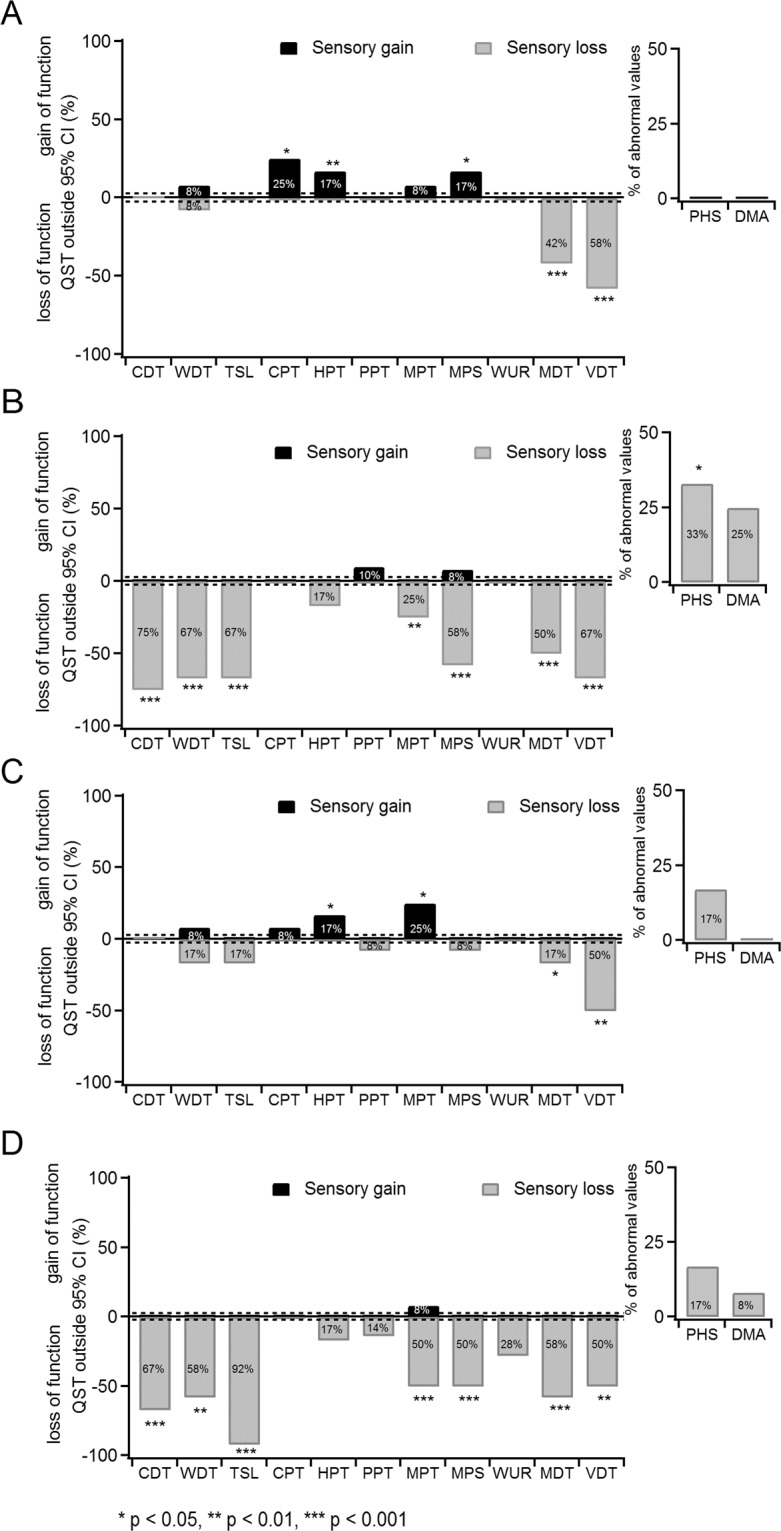
Frequencies of abnormal quantitative sensory testing (QST) parameters. Positive values indicate positive sensory signs (black bars, hyperaesthesia and hyperalgesia), whereas negative values indicate negative sensory signs (light grey bars, hypoaesthesia and hypoalgesia). Dashed lines: expected range for able-bodied participants (±2.5%). Insets show values for PHS and DMA parameters. Significance when compared to the normative values [23]: *p < 0.05, **p < 0.01, ***p < 0.001 (One-way *t*-test). **a** Control site (hand) QST parameters in SCI NeP (*n* = 12) **b** Test site (trunk, thigh or foot) QST parameters in SCI NeP. **c** Control site (hand) QST parameters in SCI no NeP (*n* = 12) **d** Test site (trunk, thigh or foot) QST parameters in SCI no NeP. SCI spinal cord injury, NeP neuropathic pain, CDT cold detection threshold, WDT warm detection threshold, TSL thermal sensory limen, CPT cold pain threshold, HPT heat pain threshold, PPT pressure pain threshold, MPT mechanical pain threshold, MPS mechanical pain sensitivity, WUR wind-up ratio, MDT mechanical detection threshold, VDT vibration detection threshold, PHS paradoxical heat sensations, DMA dynamic mechanical allodynia, CI confidence interval.

Figure 1 shows the frequencies of abnormal values in SCI NeP and SCI no NeP groups. Abnormal sensory loss was prevalent at the test site, comprising thermal detection, pain parameters and mechanical detection. There was an increased incidence of paradoxical heat sensations (PHS) (4/12 (33%)) and dynamic mechanical allodynia (DMA) (3/12 (25%)) at the test site in SCI NeP group, which coincided with the presence of burning pain sensations evaluated with NPSI subscore ≥7.

Remarkably, both groups with SCI had abnormalities also at the control site (Table 3; Fig. 1), which were expressed in frequent loss of function for VDT (7/12 (58%) in SCI NeP and 6/12 (50%) in SCI no NeP). Both groups with SCI also showed sensory gains in QST parameters: (1) SCI NeP in cold pain threshold (CPT) (3/12 (25%)), heat pain threshold (HPT) (2/12 (17%)), mechanical pain threshold (MPT) (1/12 (8%)) and mechanical pain sensitivity (2/12 (17%)) and (2) SCI no NeP in WDT (1/12 (8%)), CPT (1/12 (8%)), HPT (2/12 (17%)) and MPT (3/12 (25%)). In addition, SCI NeP group had more cases (5/12, 42%) of loss of function in MDT than SCI no NeP (2/12, 17%).

### Laser-evoked potentials

**Fig. 2:**
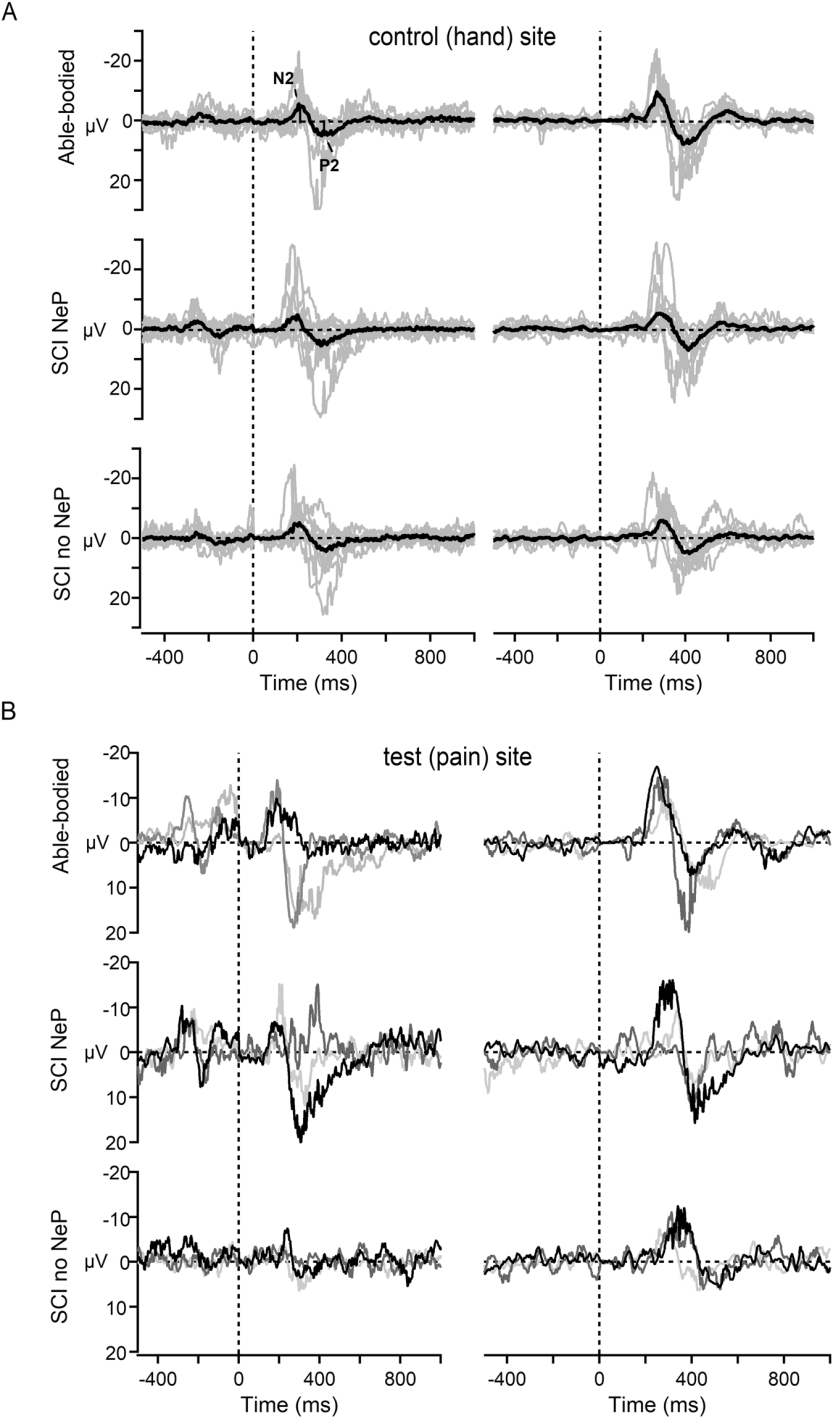
Summary of late laser-evoked (LEPs) and contact heat-evoked potentials (CHEPs). **a** Grand averages (black traces) and individual average LEPs (left panel, grey traces) and CHEPs (right panel, grey traces) waveforms from the control site (hand) measured in three groups: able-bodied (upper traces), SCI NeP (middle traces) and SCI no NeP (lower traces). Negative values are plotted upwards. Statistical mean values of the LEPs and CHEPs N2 and P2 latencies as well as N2P2 aplitudes were not different between the groups (*p* > 0.05, One-way ANOVA or Kruskal–Wallis one-way ANOVA on ranks). **b** Examples of LEPs (left panel) and CHEPs (right panel) waveforms measured from the test site (trunk, thigh or foot) from those individuals in SCI NeP (middle traces) and SCI no NeP (lower traces) groups, in whom LEPs and CHEPs were preserved (*n* = 3 for each group). The upper traces show examples of individual LEPs (left panel) and CHEPs (right panel) traces from three matching able-bodied participants. SCI spinal cord injury, NeP neuropathic pain, N2 negative component N2, P2 positive component P2.

The Aδ-fibres stimulation paradigm reliably evoked LEPs on the control site in 9/12 SCI NeP, 8/12 SCI no NeP and 11/12 able-bodied participants, which did not differ between the groups and were within the normative values [25] (Supplementary Table 1, Fig. 2A). In contrast to able-bodied group, where LEPs on the test site were evoked in 10/12 participants, only 3/12 participants showed evoked responses in each group with SCI (Supplementary Table 2). Parameters of evoked responses in all groups on the test site were within the normative values. The examples of LEPs from these three participants are shown on the Fig. 2B together with matched traces from test sites in able-bodied participants. Sometimes, there was an artefact on LEPs at around −250 ms during the 500 ms period before stimulus, because of internal signal processing procedure from the mechanical pedal movement to the optical output. The pain ratings (with NRS) following each LEP stimulus showed no difference between all three groups at the control site and were perceived as more painful by the able-bodied group than by the other two groups with SCI at the test site (Supplementary Tables 3 and 4).

### Reaction time analysis

**Fig. 3:**
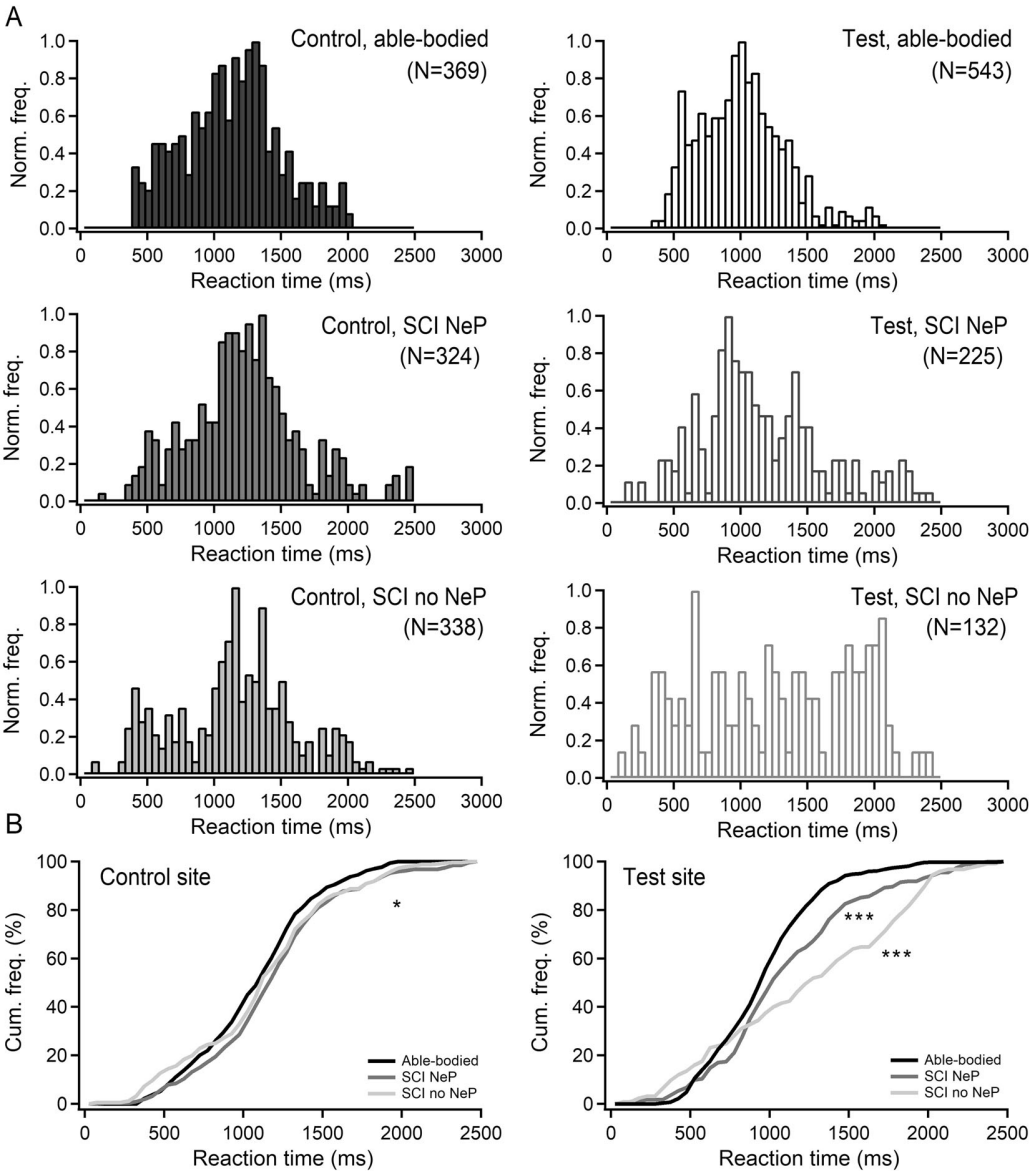
Reaction times of ultra-late laser-evoked potentials (ULEPs) inciting stimuli. **a** (left panel) Reaction time (RT) distribution analysis for the control (left column) and test (right column) sites in all three groups: able-bodied participants (first panel), SCI NeP participants (second panel) and SCI no NeP participants (third panel). **b** Cumulative histogram plots for the able-bodied (black line), SCI NeP (dark grey) and SCI no NeP (light grey) groups. Both groups with SCI showed difference in RT distribution at the control (hand, *p < 0.05, Kolmogorov–Smirnoff test) and test sites (***p < 0.001, Kolmogorov–Smirnoff test) in comparison to the able-bodied group. SCI spinal cord injury, NeP neuropathic pain, Cum. freq. cumulative frequency, Norm. freq. normalized frequency.

The RT analysis at the control site showed that laser stimuli were perceived in 88% trials by the able-bodied group, in 77% by the SCI NeP and in 80% by the SCI no NeP. Normalized histograms of perceived stimuli on the control site showed bimodal RT distributions for all groups (Fig. 3A, left panel) with the first peak at 250–600 ms (Aδ-fibres activation) and the second peak at 1000–1500 ms (C-fibres activation). Overall, there was a difference in RT distributions between able-bodied and both groups with SCI (*p* < 0.05, Kolmogorov–Smirnoff test). The differences between mean RT values in three groups (able-bodied: 1100 ms (95% CI: 1061–1139); SCI NeP: 1222 ms (95% CI: 1170–1274); SCI no NeP: 1161 ms (95% CI: 1061–1139)) were tested with Kruskal–Wallis test for three independent samples revealing significant difference (*p* = 0.007, H_2, 1031_ = 9.89). Post-hoc pairwise Bonferroni-corrected comparison showed significant difference only between able-bodied and SCI NeP groups (*p* = 0.005).

The RT analysis at the test site showed that stimuli were perceived in 75% trials by the able-bodied group, but only in 31% by the SCI NeP and 18% by the SCI no NeP. Normalized histograms of perceived stimuli on the test site from able-bodied and SCI NeP group showed RT distributions only with one peak around 1 s corresponding to C-fibres activation (Fig. 3A, right panel). In contrast, RTs on the test site in SCI no NeP group was scattered without clear peaks. There was statistical significance between all three groups (*p* < 0.001, Kolmogorov–Smirnoff test). These differences in RT distributions were also represented in the cumulative histogram plots (Fig. 3B). The differences between mean RT values in three groups (able-bodied: 988 ms (95% CI: 952–1010); SCI NeP: 1135 ms (95% CI: 1071–1199); SCI no NeP: 1325 ms (95% CI: 1210–1440)) were tested with Kruskal–Wallis test for three independent samples revealing significant difference (*p* = 0.000, H_2, 900_ = 37.52). Post-hoc pairwise Bonferroni-corrected comparison showed significant difference between all three groups: able-bodied and SCI NeP (*p* = 0.001); able-bodied and SCI no NeP (*p* = 0.000); SCI NeP and SCI no NeP (*p* = 0.05).

### Contact heat-evoked potentials

Similar to LEPs, CHEPs of the control site evoked clear Aδ-mediated cortical potentials in all three groups, which did not differ between the groups and were within the normative values [26] for both latencies and amplitudes (Supplementary Table 5, Fig. 2A). In contrast, CHEPs recorded from the test site were absent in 9/12 participants in both groups with SCI in comparison to the able-bodied group (Supplementary Table 6). If CHEPs were preserved, both N2/P2 components were within the normative values. Evoked potentials from the corresponding test sites, which were preserved in three participants in each SCI group, were shown on Fig. 2B together with three matched participants from able-bodied group.

## Discussion

In this study, we used multi-dimensional approach to investigate participants with SCI with and without NeP and compared them to the able-bodied matched individuals. Both groups with SCI had only minor differences in the clinical characteristics thus it should have a marginal impact on the presented results. Indeed, it was shown before, that there was no relationship between the presence of pain overall and the level of lesion, completeness or type of injury [3].

QST revealed altered spinothalamic and dorsal column functions below the NLI in both groups with SCI unspecific to the NeP, which goes in line with the previous studies [27–29]. Electrophysiological investigations in our study, using LEPs and CHEPs were equally able to detect lesion in spinothalamic tract (STT) after SCI, but could not provide the information about the degree of somatosensory impairment. These results confirmed a great similarity between these two techniques [30], which could be used by clinical setting, depending on what is available and taking into account that CHEPs could be more painful than LEPs as was observed in our study. Some abnormalities were also found in dorsal column functions above the NLI (at the control site) as compared to the able-bodied participants. Such sensory changes in unaffected (ipsilesional body area) side have been demonstrated by QST in individuals with stroke, reflecting chronic maladaptive cortical plasticity [31].

Although participants with SCI in our study represented unselected patient cohort with relatively mild NeP symptoms, we could still observe some differences within the SCI NeP group with several participants who had more severe NeP pain symptoms (intensity of burning pain NPSI subscore ≥7) and gain in PHS and/or DMA parameters of QST. Indeed, it was shown that mechanical and/or thermal allodynia can be associated with the NeP development in patients with SCI [8, 32] and has prevalence of 47% in patients with below-level pain. Previous studies suggested that these results might point towards the signs of sensitisation, which is not related to the SCI by itself, but rather reflects secondary processes that might cause disinhibition and central sensitisation in STT neurons [7, 8, 28, 33, 34]. Particularly, the role of C-fibres has been addressed. Investigations in participants with SCI with and without NeP by using activation and sensitisation of C-fibres with the combination of heat and/or cold QST stimuli and topical capsaicin, showed neuropathic-like pain sensations only in SCI NeP participants [35]. Authors suggested that residual hypersensitized C-nociceptor fibres within the lesioned STT pathways could distinguish people with central pain from those without. Recent reviews showed that central sensitisation of nociceptors is also prominent in other patient cohorts, for example with small fibre neuropathies, osteoarthritis, musculoskeletal disorders and headache [36–41]. The degree to which this sensitisation and consequently pain can be aggravated could be influenced by genetic and environmental factors and such comorbid conditions as anxiety, depression or medication overuse.

In this study, we also explored C-fibres function using thulium laser. We observed that RTs were longer when compared groups with SCI to the able-bodied group both on control and test sites. The fact that RTs were also abnormal on the control site in SCI suggests some changes in STT above the NLI as compared to able-bodied individuals. In addition, RT distribution in SCI no NeP on test site was scattered and without clear peak in contrast to SCI NeP, pointing towards higher degree of C-fibre preservation in the latter group.

Such delayed responses to selective C-fibre stimulations in participants with SCI might indicate either single or compound changes in the peripheral sub-modalities of somatosensory system, transmission on the spinal cord level or central processing. It was shown in animal models of SCI injury that following SCI, spontaneous activity initially developed at the site of lesion, could eventually progress both to the periphery and to higher levels of central nervous system, leading to alternations in neuronal signalling not only in injured, but also in uninjured C-fibres [42]. These uninjured C-fibres could develop enhanced responsiveness to natural stimuli and one of the proposed mechanisms for this phenomenon was activity-dependent slowing of the fibres due to decrease in hyperpolarization-activated inward current (*I*_h_), which represents important modulator of action potential firing frequency in many excitable cells [43]. Similar observations were shown in humans from studies of pathological C-fibres with microneurographic recordings in patients with erythromelalgia [44] and various peripheral neuropathies [45]. The alternations were demonstrated in both heat- and mechano-sensitive (“polymodal”) and mechano-insensitive (“silent”) C-fibres. Sensitization and spontaneous activity were shown in mechano-insensitive C-fibres. Decreased conduction velocity and increased activity-dependent slowing was found in polymodal C-fibres. In our study, laser thermal heat stimulations evoked primarily polymodal C-fibres, which delayed RTs in participants with SCI in comparison to able-bodied individuals could be also explained by increased level of excitation and decreased conduction velocity as suggested in above-mentioned studies [44, 45]. Interestingly, similar results were also obtained in depressed patients, who had longer RTs and increased pain threshold levels than able-bodied people [16]. Indeed, SCI NeP participants in our study showed elevated levels of depression in comparison to able-bodied individuals and those without NeP, confirming previous studies [3]. The influence of depression on QST parameters has also been discussed [46]. This observation could either support the role of depression in pain chronification or might be one of the limitations of the study.

This study had also other limitations. First, for technical reasons we excluded participants who could be relevant to our study. For example, we included only participants with lesion below T1, because the acquisition of QST results was dependent of an intact sensory-motor hand function to deal with a computer mouse. In addition, we only include participants with partially persevered sensation in the testing area, because in completely deafferentiated area LEPs, ultra-late LEPs, CHEPs and QST would not show a meaningful result. Second, participants with different underlying pathology of the paraplegia were included either due to spinal cord lesion, cauda equina lesion or both. However, it is unknown whether these participant groups show different pain presentations and/or pain-related neurophysiology as LEPs, CHEPs or QST. Therefore, further research is needed to evaluate potential differences in these groups or between participants with neuropathic at-level and below-level SCI pain. Third, it cannot be excluded that significant difference for longer time since injury in the SCI no NeP group might be a limitation, but according to Sidall et al. 2001 [3] and Jensen et al. 2007 [47], NeP in SCI is a chronic phenomenon, which probably will not go away with time. Fourth, clinical applicability of ultra-late LEPs in assessing patients with SCI remains restraint and further studies are necessary to improve this technique. In addition, studies should be done in larger population and, preferably, without pain medication, often used by patients with SCI NeP, which could considerably influence results and may bias the study.

## Conclusions

Changes in somatosensory functions were detected below the level of injury independent of NeP. Additional changes at the control site indicate modifications in sensory processing rostral to the spinal lesion. There were no differences according to the presence of NeP in Aδ-mediated cortical potentials: late LEPs and CHEPs. However, persons with SCI and NeP demonstrated more preserved responses compared to those without NeP in C-fibre mediated RT analysis. Those results suggest that RT analysis during laser stimulation could be an essential component when investigating the NeP related somatosensory changes in persons with SCI.

## Supplementary information

Supplementary file

## Data Availability

The datasets generated during the current study are available from the corresponding author upon request.
