# Supplementary material for: Multimodal sensory evaluation of neuropathic spinal cord injury pain: an experimental study
Source: Spinal Cord. 2021 Jan 14;59(8):842–54. doi: 10.1038/s41393-020-00607-z (PMC8338558; doi:10.1038/s41393-020-00607-z)
Supplement: Supplementary file 1 — Supplementary file [file 41393_2020_607_MOESM1_ESM.pdf]

Authors: Emmanuelle Opsommer, Natalya Korogod, Lenka Stockinger, Gunther Landmann

Title: Multimodal sensory evaluation of neuropathic spinal cord injury pain: an experimental study.

Spinal Cord, 2020

Supplementary file 1 provides additional information on experimental procedures. Supplementary figures provide results of screening procedure and, individual and average quantitative sensory testing (QST) profiles at the control and test sites in participants with spinal cord injury (SCI). Supplementary tables provide additional information on evoked-potentials data.

Supplementary file 1: Experimental procedure

The intraclass correlation coefficient (ICC) analysis, performed on two trials of ultra-late laser-evoked potentials (ULEPs) measurements on the control site in able-bodied participants, confirmed that the sample size of at least 12 participants is enough in each group to have reliable measurements and ensure that the lower limit of 95% confidence interval (CI) for ICC=0.9 is no less than $\rho_0=0.6$ with 80% assurance probability, which is considered acceptable.

All experiments were performed in an experimental room with temperature kept constant (between 21-23°C) and a degree of humidity of 50-60%.

Participants were familiarized with the stimulation and recording procedure during a test session. Clinical examinations, (QST), contact heat-evoked potentials (CHEPs), laser-evoked potentials (LEPs) and reaction time (RT) measurements were performed within one day.

All tests were done on participants in supine position. Participants wore protective goggles for security reasons. For evoked potentials, participants fixed a point in front of them to minimize eye movements, to do not squeeze teeth and to relax before each stimulus. They also wore headphones with “white noise” to avoid any acoustic interference during the stimulation, but still able to perceive the human voice to follow instructions. After a command given by the examiner, participant was asked

to give pain ratings on a numerical rating scale, briefly blink eyes and again stare at a fix point [12]. This procedure was repeated until all 20 stimulus were given. We applied 2x20 stimuli to each stimulation site, except for the test site on a tiny stimulation area for ULEPs, where we used 3x20 stimuli. Receptor fatigue or sensitization was avoided by slightly moving the laser beam between each stimulus.

For LEPs, the analyzed time window of 4000ms included a fore period of 500ms before the stimulus onset. Trials contaminated by artefacts were rejected after visual inspection for subsequent analysis. For each participant, average LEP waveforms were computed for each stimulus type and each stimulus location. Laser-evoked potential components, such as latencies of the negative N2 and positive P2 peaks (ms, measured from stimulus onset to peak) as well as N2/P2 amplitude (µV, peak to peak) were obtained using a neurophysiology machine, type VikingSelect and version 12 (Nicolet Biomedical Inc.). Data were stored on disk for off-line analysis.

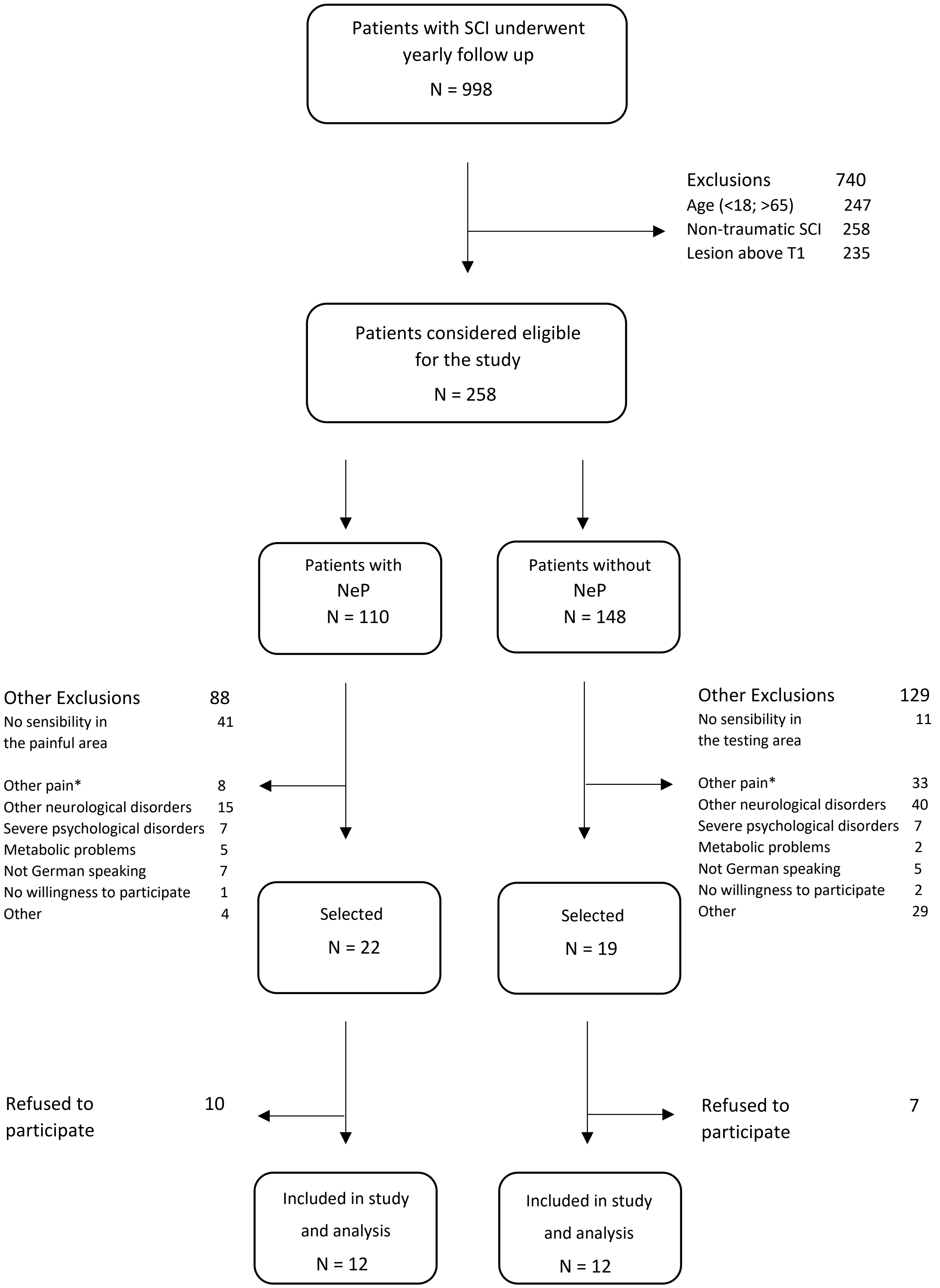
Patients with SCI underwent yearly follow up
N = 998
Exclusions 740
Age (<18; >65) 247
Non-traumatic SCI 258
Lesion above T1 235
Patients considered eligible for the study
N = 258
Patients with NeP
N = 110
Patients without NeP
N = 148
Other Exclusions 88
No sensibility in the painful area 41
Other pain* 8
Other neurological disorders 15
Severe psychological disorders 7
Metabolic problems 5
Not German speaking 7
No willingness to participate 1
Other 4
Other Exclusions 129
No sensibility in the testing area 11
Other pain* 33
Other neurological disorders 40
Severe psychological disorders 7
Metabolic problems 2
Not German speaking 5
No willingness to participate 2
Other 29
Selected
N = 22
Selected
N = 19
Refused to participate 10
Refused to participate 7
Included in study and analysis
N = 12
Included in study and analysis
N = 12

Supplementary Figure 1. Flow chart of the patients screening procedure. * “Other pain” meaning for groups with SCI: nociceptive pain below the lesion level, other neuropathic pain, other pain or unknown pain corresponding to ISCIP classification (Bryce et al., 2012b).

Abbreviations: NeP, Neuropathic Pain; SCI, Spinal Cord Injury; ISCIP classification, International Spinal Cord Injury Pain Classification.

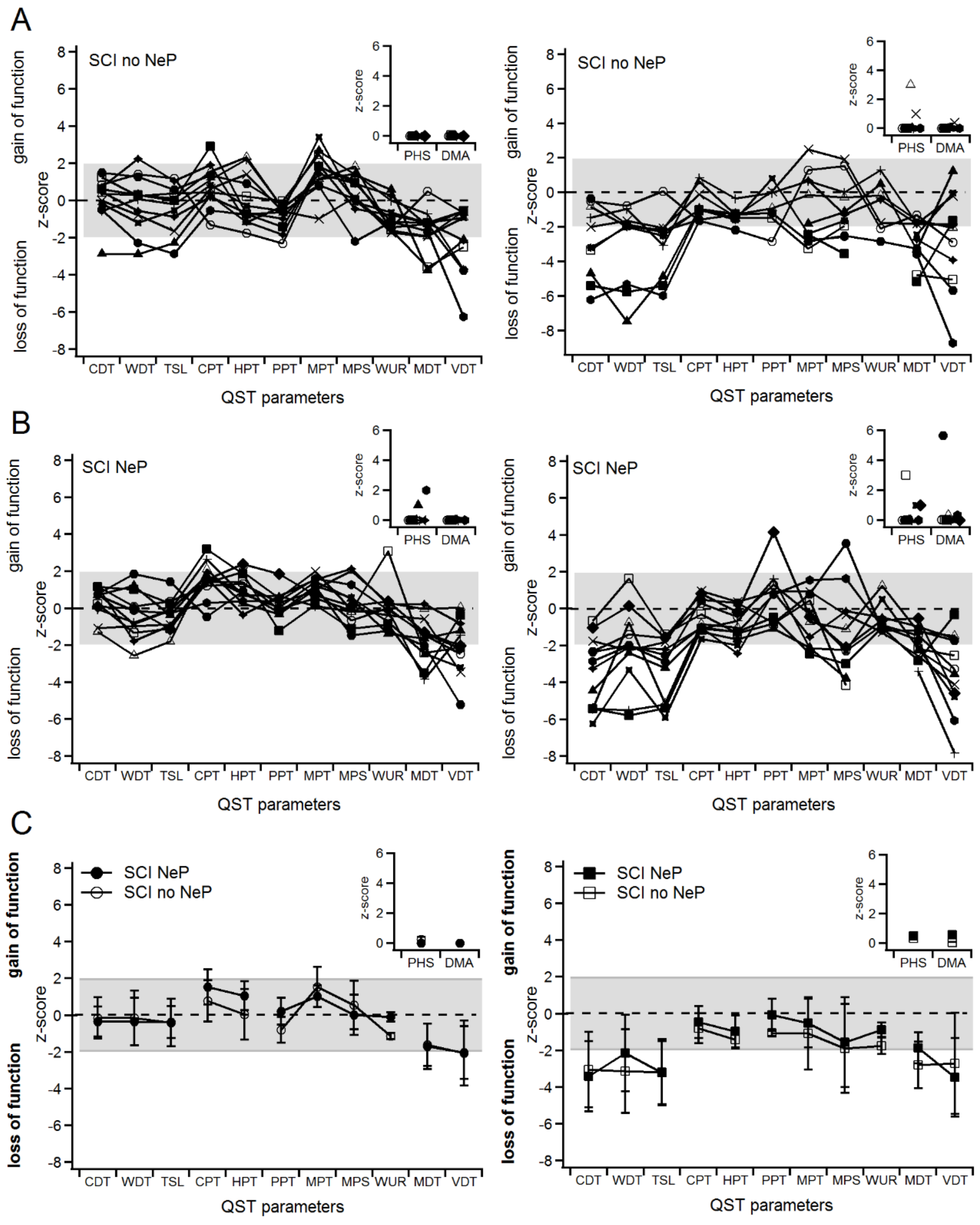

Supplementary Figure 2. Individual (*A*, *B*) and average (*C*) quantitative sensory testing (QST) profiles at the control (hand, *left panel*) and test (*right panel*) sites in SCI no NeP (*A*,*C*) and SCI NeP (*B*, *C*) groups. Positive z scores indicate positive sensory signs (hyperaethesia and hyperalgesia), whereas negative z scores indicate negative sensory signs (hypoaesthesia and hypoalgesia). Grey area: 95% confidence interval for able-bodied participants (-1.96 < z < 1.96). Insets show individual average z score values

for PHS and DMA. In general, participants with SCI from both groups showed similar z-score profiles both on the control and test sites. However, there were more cases of abnormal values for PHS and DMA in SCI NeP than in SCI no NeP.

Abbreviations: SCI, spinal cord injury; NeP, neuropathic pain; CDT, cold detection threshold; WDT, warm detection threshold; TSL, thermal sensory limen; CPT, cold pain threshold; HPT, heat pain threshold; PPT, pressure pain threshold; MPT, mechanical pain threshold; MPS, mechanical pain sensitivity; WUR, wind-up ratio; MDT, mechanical detection threshold; VDT, vibration detection threshold; PHS, paradoxical heat sensations; DMA, dynamic mechanical allodynia.

Supplementary Table 1. Late laser-evoked potential components' values (mean ± standard deviation, n= number of participants concerned by the values) recorded from the control site (hand).

Parameter	SCI NeP	SCI no NeP	Able-bodied	P-value
LEPs latency N2 (ms)	211 ± 41 (n=9)	220 ± 31 (n=8)	228 ± 22 (n=11)	0.530[¥]
LEPs latency P2 (ms)	293 ± 45 (n=9)	323 ± 66 (n=8)	328 ± 42 (n=11)	0.294[¥]
LEPs amplitude N2P2 (µV)	25 ± 15 (n=9)	22 ± 13 (n=8)	17 ± 12 (n=11)	0.326[#]

Abbreviations: SCI: spinal cord injury. NeP: neuropathic pain. LEPs: laser-evoked potentials. N2: negative component N2. P2: positive component P2. [¥]One Way ANOVA. [#]Kruskal Wallis One Way ANOVA on ranks.

Supplementary Table 2. Late laser-evoked potential components' values (mean ± standard deviation, n= number of participants concerned by the values) recorded from the test site.

Parameter	SCI NeP	SCI no NeP	Able-bodied
LEPs latency N2 (ms)	263 ± 107 (n=3)	231 ± 26 (n=3)	215 ± 24 (n=10)
LEPs latency P2 (ms)	351 ± 75 (n=3)	296 ± 42 (n=3)	323 ± 27 (n=10)
LEPs amplitude N2P2 (µV)	23 ± 7 (n=3)	12 ± 3 (n=3)	21 ± 7 (n=10)

Abbreviations: SCI: spinal cord injury. NeP: neuropathic pain. LEPs: laser-evoked potentials. N2: negative component N2. P2: positive component P2.

Supplementary Table 3. Evoked potentials NRS pain values for the control site.

Parameter	SCI NeP	SCI no NeP	Able-bodied	P-value
LEPs NRS	1.8 ± 1.3	2.1 ± 1.6	2.2 ± 1.8	0.271[#]
CHEPs NRS	2.3 ± 1.7	3.2 ± 2.2	4.2 ± 2.1	<0.001[#]

Abbreviations: SCI: spinal cord injury. NeP: neuropathic pain. LEPs: laser-evoked potentials. CHEPs: contact heat-evoked potentials. NRS: numerical rating scale. [#]Kruskal Wallis One Way ANOVA on ranks.

Supplementary Table 4. Evoked potentials NRS pain values for the test site.

Parameter	SCI NeP	SCI no NeP	Able-bodied	P-value
LEPs NRS	1.1 ± 1.4	1.3 ± 2.0	3.1 ± 1.8	<0.001[#] (Able-bodied-SCI NeP; Able-bodied-SCI no NeP)
CHEPs NRS	1.7 ± 1.7	2.0 ± 2.3	4.9 ± 2.4	<0.001[#] (Able-bodied-SCI NeP; Able-bodied-SCI no NeP)

Abbreviations: SCI: spinal cord injury. NeP: neuropathic pain. LEPs: laser-evoked potentials. CHEPs: contact heat-evoked potentials. NRS: numerical rating scale. [#]Kruskal Wallis One Way ANOVA on ranks.

Supplementary Table 5. Contact heat-evoked potential components' values (mean ± standard deviation, n= number of participants concerned by the values) recorded from the control site.

Parameter	SCI NeP	SCI no NeP	Able-bodied	P-value
CHEPs latency N2 (ms)	302 ± 33 (n=10)	301 ± 35 (n=10)	282 ± 19 (n=10)	0.243[¥]
CHEPs latency P2 (ms)	399 ± 32 (n=10)	412 ± 45 (n=10)	406 ± 29 (n=10)	0.733[¥]
CHEPs amplitude N2P2 (µV)	24 ± 15 (n=10)	20 ± 7 (n=10)	26 ± 11 (n=10)	0.465[#]

Abbreviations: SCI: spinal cord injury. NeP: neuropathic pain. CHEPs: contact heat-evoked potentials. N2: negative component N2. P2: positive component P2. [¥]One Way ANOVA. [#]Kruskal Wallis One Way ANOVA on ranks.

Supplementary Table 6. Contact heat-evoked potential components' values (mean ± standard deviation, n= number of participants concerned by the values) recorded from the test site.

Parameter	SCI NeP	SCI no NeP	Able-bodied
CHEPs latency N2 (ms)	322 ± 16 (n=3)	327 ± 19 (n=3)	290 ± 27 (n=11)
CHEPs latency P2 (ms)	405 ± 8 (n=3)	437 ± 21 (n=3)	422 ± 30 (n=11)
CHEPs amplitude N2P2 (µV)	20 ± 9 (n=3)	15 ± 2 (n=3)	23 ± 8 (n=11)

Abbreviations: SCI: spinal cord injury. NeP: neuropathic pain. CHEPs: contact heat-evoked potentials. N2: negative component N2. P2: positive component P2.
